# The Effect of Polymer Powder on the Cracking of the Subbase Layer Composed of Cold Recycled Bitumen Emulsion Mixtures

**DOI:** 10.3390/ma14195867

**Published:** 2021-10-07

**Authors:** Jakub Krasowski, Przemysław Buczyński, Marek Iwański

**Affiliations:** Department of Transportation Engineering, Faculty of Civil Engineering and Architecture, Kielce University of Technology, Al. Tysiąclecia Państwa Polskiego 7, 25-314 Kielce, Poland; p.buczynski@tu.kielce.pl (P.B.); iwanski@tu.kielce.pl (M.I.)

**Keywords:** cold-recycled mixture, bitumen emulsion, polymer, SCB, subbase, fracturing

## Abstract

The research was aimed at assessing the effect of the redispersible polymer powder on the fracture resistance of a subbase made of a mineral–cement mixture with a bitumen emulsion. The test was performed at two temperatures, i.e., 0 °C and 20 °C. The prepared mixtures differed in the content of cement, asphalt emulsion, and polymer modifier. Cement and redispersible polymer powder were dosed in 1.5% steps from 0.5% to 3.5% while the amount of bitumen emulsion ranged from 0.0% to 5.0%. The SCB (semi-circular bending) tests carried out in the laboratory showed the dependence of the influence of the amount of binder and polymer modifier on the fracture resistance of the recycled subbase. Mixes containing a polymer modifier in their composition are characterized by a much higher resistance to cracking than traditional mineral–cement–emulsion mixtures. An example is the doubling of the framework’s fracture toughness (KIC) when the amount of the polymer modifier is increased from 0.5% to 2.0% with a constant cement content of 0.5%. The obtained results (KIC) in this case were 2.90 and 5.81. The key is the right ratio of polymer powder and cement in the base composition.

## 1. Introduction

Subbases with hydraulic binders are commonly used in pavement structures. Examples of such materials include, among others, mixtures made using the deep cold recycling technology. These can feature mineral–cement mixtures with bitumen emulsion as well as mineral–cement mixtures with foamed asphalt [1,2,3]. The road industry is seeking new solutions that can provide more favorable quality parameters of the indicated mixtures compared to the traditional ones. There have been attempts to modify the compositions of cold recycled mixtures, known examples are the addition of rubber crumbs [4,5], or utilizes dust by-products generated from aggregate de-dusting [6,7,8]. Mixtures with foamed asphalt are also a promising direction. An important factor is the improvement of the asphalt’s frothiness by using various additives [9,10,11]. It is also extremely important for the industry to precisely identify the stiffness modulus of recycled mixtures. This parameter is strongly affected by the quantity and quality of the hydraulic binder added to the mix, as well as the content of binders in the subbase. This topic has already been thoroughly analyzed [12,13,14,15]. If the recycled subbase stiffens excessively, there is a risk of transferred fracturing. This is fracturing that occurs in the subbase layer and is transferred throughout the entire structure’s thickness up to the wear layer. It seems to be important to test the fracture resistance of mixtures made using the deep cold recycling technology, but also of all materials used in the road pavement layers [16,17,18]. It is worth mentioning bound mixes used as Cement Bound Mixtures (CBM). These mixtures do not contain a bituminous binder, but only a hydraulic binder. Despite the fact that they can constitute a permanent and load-bearing subbase, the risk of fracturing is even higher.

The modification of materials with polymers seems to be an interesting and future-oriented direction in construction. The aforementioned materials include asphalts, cement concretes and mineral–asphalt mixes [19,20,21]. Łukowski conducted a thorough analysis of the impact of polymers on cement mortars and concrete in his work [22]. This constituted a signal for the attempt to use polymer to modify recycled mixtures. Buczyński was the first person to take action in this aspect, which resulted in published works [23,24]. The subject literature also features a paper in which the authors used polymer to modify the composition of a binder used for manufacturing a CBM mixture [25]. There, very interesting results were obtained, compared with the results of studies of traditional related mixtures.

The conducted literature analysis and own experiences were used in the attempt to modify the composition of a recycled mineral–cement mixture with bitumen emulsion. A polymer powder was used for the modification. The mixtures developed this way were tested in terms of their fracture resistance. The limitation of fracturing in the subbase is extremely important. It affects the durability of the subbase made using the deep cold recycling technology, thereby affecting the entire pavement structure. The results obtained in the tests suggest that the use of an adequate polymer modifier quantity in relation to cement and bitumen emulsion can substantially improve the subbase’s operating properties in the case of long-term exploitation.

## 2. Experiment Design

The conducted tests were aimed at assessing the impact of polymer powder on the reduction in fracturing in a recycled subbase made from a mineral–cement mix with bitumen emulsion (BE-CRM). In order to precisely identify the issue, it was decided to base the scope of testing on the Box-Behnken design. The design was used to select thirteen different formulae required for making BE-CRM mixtures. The mixtures differed in terms of the content of polymer powder modifier, cement, and bitumen emulsion. The polymer powder and cement were dosed with a 1.5% increment, and their content in the recycled mixture amounted to 0.5%, 2.0% and 3.5%, respectively. The bitumen emulsion was dosed with a 2.5% increment. The content of bitumen emulsion in the mixture amounted to 0.0%, 2.5%, and 5.0%, which provided a pure bitumen content of 1.5% to 3.0%. This allows for a reliable assessment of the polymer modifier’s impact on the reduction in the recycled subbase’s fracturing.

The Box–Behnken design belongs to experiment designs in which independent variables exist at three levels: −1, 0, and +1. Such designs do not include experiments in which all independent variables assume extreme values. This allows us to avoid experimentation in extreme conditions [26].

## 3. Materials and Methods

### 3.1. Materials

The composition of the cold recycled mixes with bitumen emulsion (BE-CRM) constituting the subject of the tests was designed in accordance with the guidelines [27]. Crushed aggregate and reclaimed asphalt pavement derived from the demolition of a voivodeship road were used in this project.

#### 3.1.1. Mineral Mixture

It was assumed that the mineral mixture contained in the cold recycled subbase consisted of 50% recycled asphalt pavement. A virgin aggregate VA was also used. The material used was excavated and processed in Świętokrzyskie mines. The crushed aggregate with a granulation of 0/31.5 mm constitutes 30% of the mixture. The other 20% is an aggregate with a granulation of 0/4 mm. Both mixtures meet the requirements specified in the guidelines [27]. Such component ratios ensure the proper granulation of the mineral mixture used for making the BE-CRM recycled subbase [27,28]. Table 1 presents the parameters for the aggregate with continuous granulation VA 0/31.5mm and in Table 2 for the aggregate 0/4mm.

**Figure 1:**
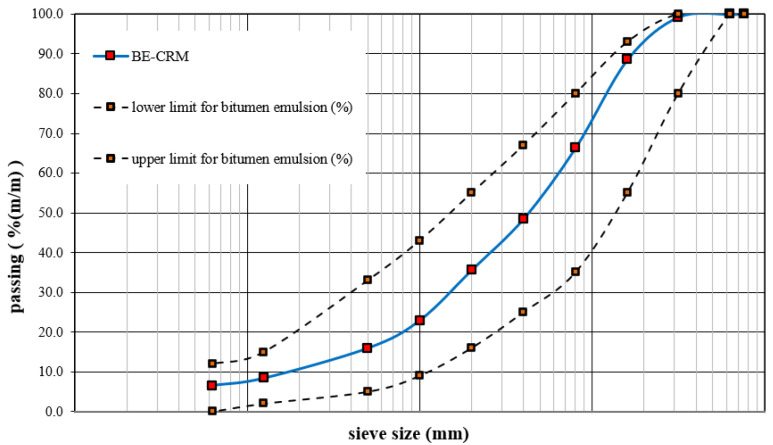
Granulation curve for the mineral mixture included in the BE-CRM.

The use of the components provided allowed for obtaining a mineral mixture with granulation that meets the requirements and fits into the limit curve values. The granulation curves are presented in Figure 1.

#### 3.1.2. Redispersible Polymer Powder

**Figure 2:**
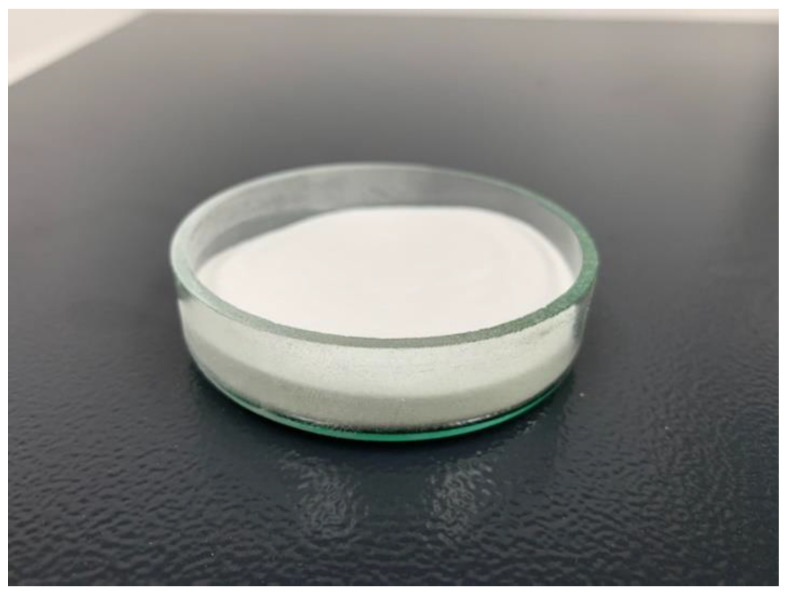
EVA redispersible polymer powder.

The BE-CRM cold recycled mixtures were made with the addition of polymer. The polymer used is a thermoplastic copolymer (polyethylene-co-vinyl octane). It occurs as a white powder generated as result of water evaporation from polymer dispersion. It is obtained from spray drying. The polymer is presented below in Figure 2. The chemical composition of the redispersible polymer powder is presented in Table 3.

Following the literature analysis [22,24], the use of the aforementioned polymer modifier in the BE-CRM may allow for the improvement of the mixture’s mechanical properties. This is due to the network connections occurring between the particles that create a continuous polymer phase [22].

The role of polymer particles in the mixture is very similar to that in cement concrete. The polymer first disperses in the liquid phase of the cement paste [22]. During the hydration process, polymer particles appear on the surface of cement grains and aggregates. The dispersed polymer particles are also trapped in the capillary pores of the aggregate. After the water evaporates, they form dense agglomerates.

During the drying of the mixture, the polymer coalesces, i.e., individual particles stick together and form a continuous polymer film. Modifying concrete mixtures with polymers brings many advantages [22]. One of the benefits is the increase in water resistance and workability of the modified mixture. Mixtures made of polymers are also characterized by lower stiffness, which translates into higher tensile and bending strength. Improved adhesion to aggregate particles increases the cohesion of the mixture. A characteristic feature of the continuous polymer layer is the bridging of voids between the aggregate particles. This ability is due to the excellent adhesion between the polymer and the mineral surfaces in the mixture.

#### 3.1.3. Cement

The BE-CRM mixtures with a polymer modifier were made using class I Portland cement with the strength of 42.5 MPa, high early strength “R” determined in accordance with EN 197-1 [37]. Binder class was selected due to the lack of additives which would possibly influence the results obtained during the recycled subbase’s testing. The basic properties of the hydraulic binder used are presented below in Table 4.

#### 3.1.4. Bitumen Emulsion

The composition of the analysed BE-CRM mixtures included a slow-breaking cationic bitumen emulsion designated as C60B10 ZM/R. The emulsion was manufactured based on bitumen 70/100. The bitumen emulsion meets the requirements specified in [41]. The basic parameters describing the binder used are presented in Table 5.

#### 3.1.5. Specimen

The aforementioned materials were used to obtain test specimens prepared in laboratory conditions. The test mixtures were prepared under laboratory conditions. Using a WLB10 laboratory mixer, the components were mixed to obtain homogeneous blends. The single batch size was 35 kg. Before the test, the optimal amount of water in the mineral mixture (OMC (Optimum Moisture Content)) was determined in accordance with the guidelines contained in EN 13286-2 [42] using the Proctor method. The OMC value was 7.0%.

The specimens made in the laboratory were compacted in accordance with the requirements specified in the test method. The basic physical and mechanical properties (i.e., bulk density, water absorption, void content, and intermediate tensile strength) were determined on specimens prepared with a Marshall hammer and dedicated perforated forms [43,44]. The compaction process requires the use of 75 blows per side at a rate of 60 blows per minute.

The bending strength was determined in the same manner as for bituminous mixtures, i.e., in a semi-circular bend (SCB) test. The test specimens had the diameter of 150 mm ± 1 mm, height of 75 mm ± 1 mm, and thickness of 50 mm ± 1 mm. The specimens were prepared for the specified test using a gyratory shear compacting press [16,45]. A number of press rotations was to ensure the maximum bulk density with the assumed air void content of Vm = 10.0%, the rotation angle amounted to 30.00 mrad, whereas the press pressure amounted to 600 kPa. The prepared specimens were 180 mm in height and 150 mm in diameter. Six test specimens meeting the specified requirements were cut out of each of the core specimens. For fracture initiation purposes, the specimens were incised at the base to a depth of 10 mm. On the first day after production, the specimen prepared in this way were stored in their molds at room temperature + 20 ± 5 °C. Subsequently, after taking out of the form, the samples were stored for 27 days at 40–70% humidity. Tests started after a 28-days conditioning period. The same conditioning procedure was applied regardless of the compaction method used.

### 3.2. Methods

#### 3.2.1. Basic Parameters of Mixtures

Physical and mechanical properties:

- Bulk density—saturated surface dry (SSD) (ρ_bssd_) [46];
- Air voids content (Vm) [47];
- Water absorption by weight (n_w_) [28];
- Indirect tensile strength (ITS_DRY_) [48].

#### 3.2.2. Semi-Circular Bending Test

**Figure 3:**
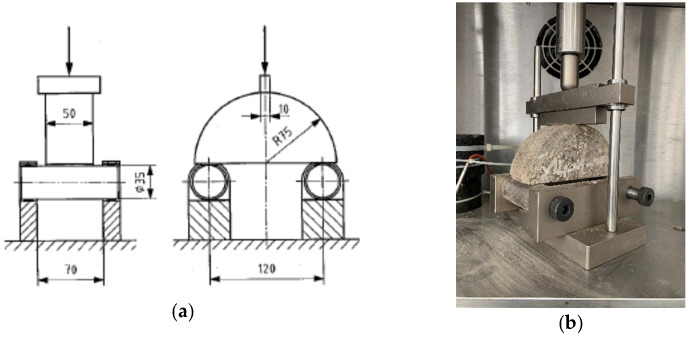
Semi-circular bending test set-up and specimen: (**a**) standard scheme (source: PN-EN 12697-44 [42]); and (**b**) fracture toughness test set-up (laboratory at Faculty of Civil Engineering and Architecture, Kielce University of Tecnology, Kielce, Poland) [49].

The material’s resistance to crack propagation was tested with a semi-circular specimen with an N notch in the base of the specimen. Notch parameters of 1.0 ± 0.10 mm of nominal width and 10.0 ± 1.0 mm of length were determined in accordance with Szydłowski’s guidelines [16]. The prepared specimen was subjected to a three-point bending test. The central part of the base side of the specimen was subjected to a tensile stress increasing at a constant strain rate of 5 mm/min. The appropriate load was increased to the maximum value of Fmax, which is directly related to the fracture toughness of the sample. Figure 3 shows an example of a bend test and test specimen setup.

The maximum strain, *ε_max_*, was calculated as follows:

(1) $$\varepsilon_{max,i}=\frac{∆W_{i}}{W_{i}}\times 100\%$$

where *W_i_* is the height of specimen *i* (*i* = 1, 2, 3, and 4) (mm) and ∆*W_i_* is the vertical strain at maximum force/load on specimen *i* (*i* = 1, 2, 3, and 4) in (mm).

The maximum peak stress/the critical stress, *σ_max_*_, *i*,_ was calculated as follows:

(2) $$\sigma_{max,i}=\frac{F_{max,i}}{D_{i}\cdot t_{i}}N/mm^{2}$$

where *D_i_* is the diameter of specimen *i* (*i* = 1, 2, 3, and 4) in (mm), *t_i_* is the thickness of specimen *i* (*i* = 1, 2, 3, and 4) in (mm), and *F_max_*_,*i*_ is the maximum load on specimen *i* (*i* = 1, 2, 3, and 4) in (*N*).

The fracture toughness, *K_IC_*, of specimen *i* (*i* = 1, 2, 3, and 4) was calculated as follows:

(3) $$K_{Ic,i}=\sigma_{max,i}\cdot Y\;\cdot \;\sqrt{\pi \cdot a_{1}}N/mm^{3/2}$$

(4) $$Y=4.782-1.219\cdot \left(\frac{a_{i}}{r_{i}}\right)+0.063exp\left(7.045\cdot \left(\frac{a_{i}}{r_{i}}\right)\right)$$

where *a_i_* is the length of the crack in specimen *I* (*i* = 1, 2, 3, and 4) (mm), *σ_max_*_,*i*_ is the critical stress on specimen *i* (*i* = 1, 2, 3, and 4) (MPa), and *Y* is the normalized stress factor by Equation (4).

## 4. Results and Discussion

### 4.1. Basic Parameters of Mixtures

The basic parameters of the prepared mixtures are presented below in Table 6. The mixtures were designated as follows: Digit and letter C—cement percentage share, digit and letter P—RPP content percentage share, and digit and letter A—content percentage of pure bitumen deriving from the emulsion. The results are presented with the assumed order of 0.5% RPP, 2.0% RPP, and 3.5% RPP. Other mixture components are also presented incrementally.

The density of the mixtures prepared during the testing differs substantially. This results from the discrepancy in the densities of particular components. The polymer powder has a density of 0.5 mg/m^3^, thereby its content in the mixture substantially reduces the value. The 0.5C-2.0P-3.0A mixture has a density of 2.15 mg/m^3^, while the 2.0C-0.5P-0.0A mixture goes up to as much as 2.293 Mg/m^3^. The average density of the prepared mixtures is approx. 2.2 mg/m^3^. The variability coefficient is between 0.0% and 0.9%. On the other hand, the standard deviation is approx. 0.01.

The air void content (Vm) in the mixtures is between 8.8% and 13.9%. The lowest values were obtained for mixtures with 2.0% RPP. The most advantageous result was obtained for the mixture with no bitumen content, including only cement and polymer, i.e., 3.5C-2.0P-0.0A. The other mixture with the lowest air void content is the formula 0.5C-2.0-1.5A. The mixture had the minimum cement and bitumen content as well as 2.0% RPP. This confirms the positive impact of RPP on the recycled subbase with simultaneous reduction in the content of other binders. It is worth noting that an excessive polymer content can increase the air void content. The obtained results’ variability coefficient for the conducted analysis is between 0.7% and 5.8%. The designated standard deviation is between 0.1 and 0.7.

The mixtures’ absorptivity is an important feature for a road subbase. The most advantageous results were obtained in subbases with polymer content of 0.5–2.0%. Cement in conjunction with the polymer seal the mixture’s structure by limiting water absorption from the environment. The pure bitumen content deriving from the bitumen emulsion causes the results to vary to a lesser degree. The lowest variability coefficient was achieved by the 0.5C-0.5P-1.5A mixture and it amounted to 1.9%. The standard deviation for the analysed mixtures was between 0.06 and 0.50.

Interesting dependencies can be observed when analysing the results of indirect tensile strength ITS_DRY_. The cement content is the mixture component that affects the result to the greatest extent. It is interesting that the 2.0C-0.5P-0.0A and 2.0C-0.5P-3.0A mixtures achieved a nearly identical result. At the content of 2% cement and 0.5% of polymer, the increase in the emulsion content did not change the ITS_DRY_ of the recycled mixtures. Similar results were also observed for recycled subbases that contained 0.5C-2.0P-0.0A and 0.5C-2.0P-3.0A. Another example can be the comparison of the strengths of the aforementioned 2.0C-0.5P-3.0A and 2.0C-2.0P-1.5A mixtures. The latter mixture contained 1.5% more polymer and reduced bitumen content. As result, an increase in stiffness of approx. 10%, from 645 kPa to 715 kPa, is obtained. An increase in bitumen content from 0.0% to 3.0% with the same cement and RPP content results in a loss in strength of approx. 40%, as in the case of the 2.0C-3.5P-0.0A and 2.0C-3.5P-3.0A mixtures. The variability coefficient obtained during the testing was between 2.0% and 8.0%. The standard deviation of the obtained indirect tensile strength results depended on the strength values

### 4.2. Semi-Cicular Bending Test

The results presented in this section were obtained during laboratory testing conducted in accordance with item 3.2. based on PN-EN 12697-44 [42]. In order to identify the properties of the cold recycled mixtures with a polymer modifier more precisely, the tests were conducted in two temperatures, i.e., 0 °C and 20 °C. The significance of the impact of particular components was designated using statistical tools presented in Table 7. The convergence surfaces of particular components were also prepared for result analysis purposes. The convergence coefficient R^2^ for the analyzed results is in the range of 0.85–0.90.

#### 4.2.1. Strain at Maximum Force ε_max_

**Figure 4:**
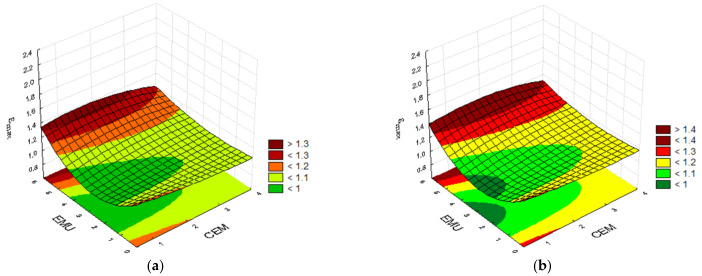

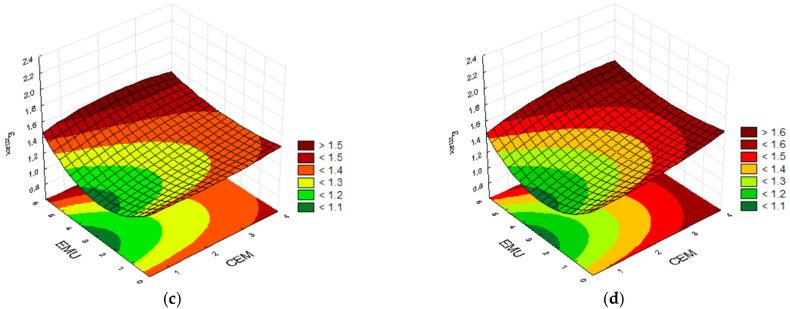
Plots for the strain at maximum force ε_max_ at the temperature of 0 °C, taking into consideration the cement and bitumen emulsion contents and the polymer modifier content of (**a**) 0.0%; (**b**) 0.5%; (**c**) 2.0%; and (**d**) 3.5%.

Several dependencies can be observed when analyzing the test results. For tests conducted at the temperature of 0°C, the highest strain value was obtained for the mixture containing 3.5% of cement, 3.5% of polymer, and 2.5% of bitumen emulsion, i.e., ε_max_ = 1.49. It is interesting that a nearly identical result, ε_max_ = 1.47, was obtained for the mixture containing 3.5% of cement, 2.0% of polymer, and 5.0% of bitumen emulsion. An interesting dependence in the form of similar results was obtained in the case of mixture containing 2.0% of cement and 3.5% of polymer modifier. One of them was prepared without using bitumen emulsion, whereas the other mixture contained 5.0% of emulsion. The former mixture achieved the strain of ε_max_ = 1.45, whereas the latter achieved ε_max_ = 1.46. This demonstrates that a proper combination of cement and polymer modifier allows us to obtain satisfactory results and that the emulsion addition does not affect the result. A similar situation occurs when the cement content is increased from 0.5% to 3.5% with a constant polymer content of 2.0% and elimination of bitumen emulsion from the composition. An increase in cement content by 3.0% results in a difference of 0.03, i.e., an increase from 1.38 to 1.41. This is almost a negligible difference despite a substantial cement content. The polymer bonds positively affect the mixture’s working nature during fracture formation. Figure 4 presents the convergence surfaces for the strain at maximum force ε_max_ at 0 °C.

The convergence surfaces depict the properties of the mineral–cement mixtures containing bitumen emulsion and polymer modifier. All plots demonstrate the dependence of the impact of cement and bitumen emulsion on the strain at maximum force ε_max_ at a constant polymer content at the temperature of 0 °C. A traditional mineral–cement–emulsion mixture is considered for 0.0% modifier content in the mixture. In such a case, the best results are obtained at the maximum emulsion content in the mixture. Similar results are obtained for the mixture containing 0.5% of polymer modifier. An increase in the recycled mixture’s polymer content to 2.0% and 3.5% allows us to obtain high strain at maximum force results with a simultaneous reduction in the emulsion content. It is therefore advantageous to slightly increase the percentage share of cement in the mixture.

**Figure 5:**
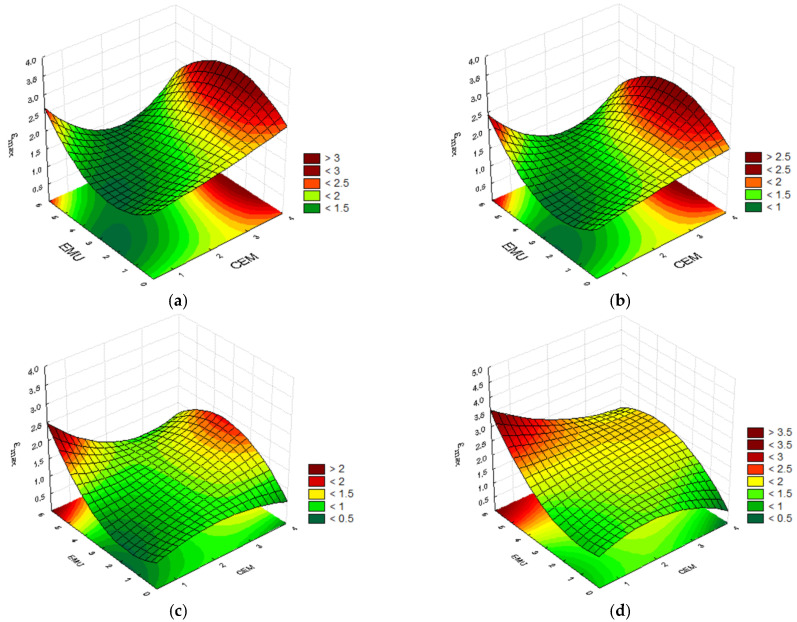
Plots for the strain at maximum force ε_max_ at the temperature of 20 °C, taking into consideration the cement and bitumen emulsion contents and the polymer modifier content of (**a**) 0.0%; (**b**) 0.5%; (**c**) 2.0%; and (**d**) 3.5%.

The mixtures’ working nature changes when the temperature is increased to 20 °C. The mixture containing 2.0% of cement, 3.5% of polymer modifier, and 5.0% of bitumen emulsion achieved one of the highest strain results. The case was similar during the test conducted at 0 °C. On the other hand, the highest strain ε_max_ = 2.22 was achieved by the recycled mixture containing 0.5% of modifier, 3.5% of cement, and 2.5% of bitumen emulsion. The results for the mixture containing 2.0% of cement, 0.5% of polymer, and 0.0% of emulsion with the ε_max_ = 1.61, as well as the mixture containing 0.5% of cement, 2.0% of polymer, and 5.0% emulsion where ε_max_ = 1.67, are also interesting. This exemplifies the impact of the binder composition optimisation and the polymer content on the mixture’s parameters. Due to the fact that the test was conducted at 20 °C, the mixtures became less stiff, and demonstrated higher susceptibility and higher strain at maximum force. Figure 5 below presents the convergence surfaces at 20 °C.

For mixtures with a polymer content of 0.0–0.5% and testing temperature of 20 °C, the highest results are obtained through a reduction in the subbase composition’s emulsion content and an increase in the cement content. In this case, the strain at maximum force ε_max_ is the highest. An increase in the polymer powder content to 2.0% and 3.5% allows us to reduce the emulsion and cement contents. This results in a substantial increase in the analyzed strain at maximum force ε_max_. The polymer has a positive impact on the mixture’s properties, enabling the generation of higher strains prior to the subbase’s fracturing.

#### 4.2.2. Stress at Break σ_max_

**Figure 6:**
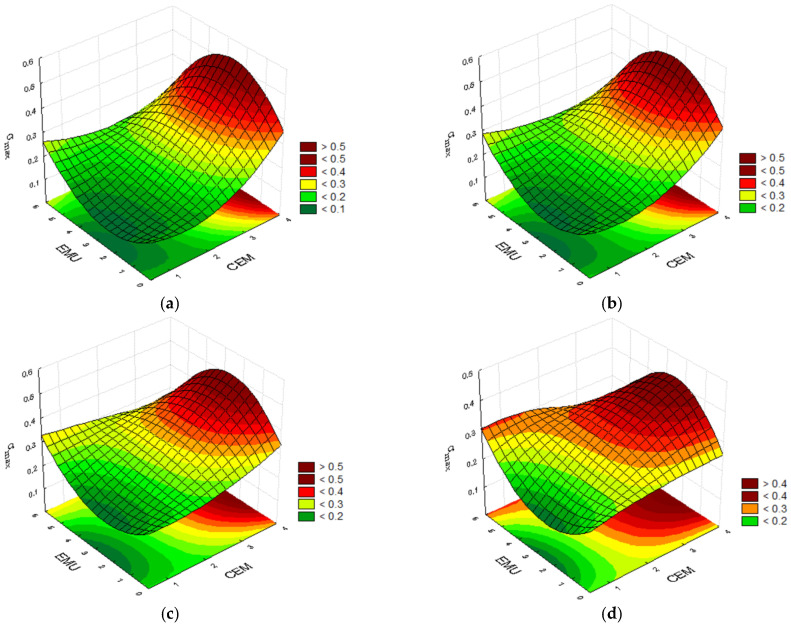
Plots for the stress at break σ_max_ at the temperature of 0 °C, taking into consideration the cement and bitumen emulsion contents and the polymer modifier content of (**a**) 0.0%; (**b**) 0.5%; (**c**) 2.0%; and (**d**) 3.5%.

The strain that occurs at the specimen’s break is the result of the mixture’s stiffness. Of course, the lower the temperature, the higher the stiffness. Therefore, the results obtained when conducting the test at 0 °C were highest in the case of recycled mixtures with the higher cement content. In the case of mixtures with 3.5% of cement content, σ_max_ amounts from 0.3 to 0.4. It is worth noting that the mixture with 2.0% of cement, 2.0% of polymer modifier, and 2.5% of bitumen emulsion achieved nearly the same result as the mixture with the same cement content as well as 3.5% of polymer content and 5.0% of bitumen emulsion content. This shows the significance of adequate binder content in the recycled subbase. This also demonstrates that there is no need to add the highest polymer and emulsion quantities to the mixture. The same result was also obtained for two other mixtures. Both mixtures contained 2.0% of cement. The first mixture contained 0.5% of polymer modifier and 5.0% of bitumen emulsion, while the other contained 3.5% of modifier and 0.0% of bitumen emulsion. The polymer allows for achieving material susceptibility without the need to use bitumen emulsion. Figure 6 below presents the convergence surfaces for the discussed parameter.

The stress at break σ_max_ test conducted at 0 °C demonstrated that the recycled mixture’s cement content is a very important parameter. The highest results were obtained for cement content of 3.5% and emulsion content of 2.0%. The addition of polymer into the mixture results in equally high stress at break σ_max_ with simultaneous reduction in cement content. Advantageous results can be obtained in a broader range of mixture component dosage.

**Figure 7:**
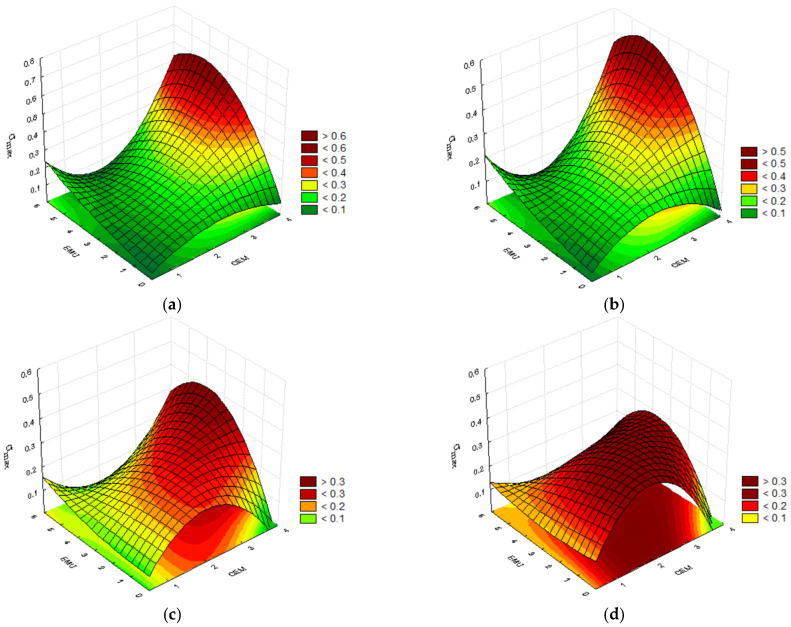
Plots for the stress at break σ_masx_ at the temperature of 20 °C, taking into consideration the cement and bitumen emulsion contents and the polymer modifier content of (**a**) 0.0%; (**b**) 0.5%; (**c**) 2.0%; and (**d**) 3.5%.

The results obtained at the temperature of 20 °C confirm the assumption. The recycled mixture containing 3.5% of cement, 3.5% of polymer, and 2.5% of bitumen emulsion achieved the same result as the mixture with 3.5% of cement, 2.0% of polymer and 5.0% of bitumen emulsion, i.e., σ_max_ = 0.29. The modifier content in the mixture can help reduce the demand for the asphalt binder. The mixture containing 2.0% of cement and 3.5% of modifier, prepared without bitumen emulsion, achieved the result of σ_max_ = 0.32. This confirms the possibility of reducing the bitumen emulsion content in the recycled subbase in favour of the modified polymer content. Figure 7 below presents the convergence surfaces for the conducted analysis.

In the recycled mixture with no polymer modifier, i.e., a typical mineral–cement–emulsion mix, the highest stress at break σ_max_ occurs at high cement contents. The addition of polymer into the recycled mixture and a gradual increase in its content allows us to obtain higher and more uniform results of stress at break σ_max_ in a test conducted at 20 °C, especially in the case of polymer modifier content of 3.5%. An increase in the modifier content thereby allows us to reduce the cement and bitumen emulsion contents in the mixture without deteriorating the material’s property, while maintaining the required durability.

#### 4.2.3. Fracture Toughness K_IC_

**Figure 8:**
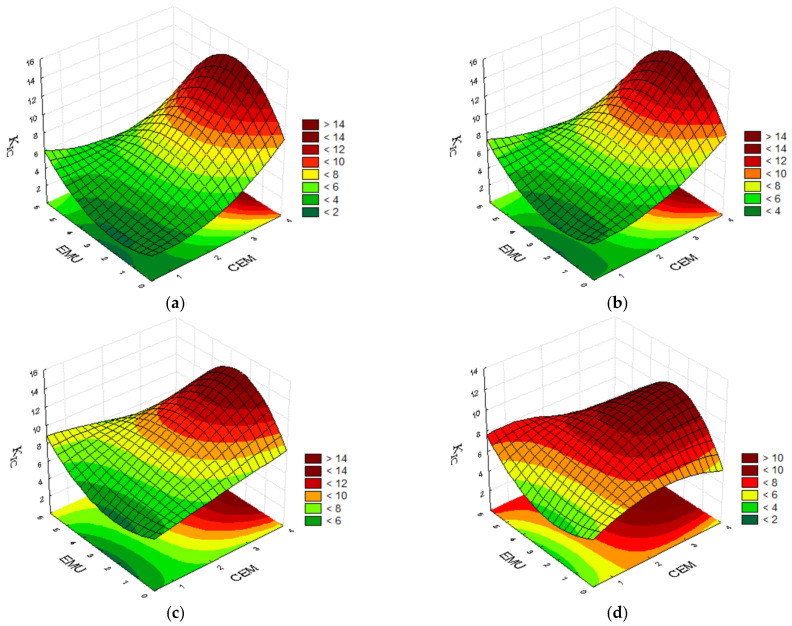
Plots for fracture toughness K_IC_ at the temp. of 0 °C, taking into consideration the cement and bitumen emulsion contents and the polymer emulsion content of (**a**) 0.0%; (**b**) 0.5%; (**c**) 2.0%; and (**d**) 3.5%.

The fracture toughness is an important feature in the case of a road subbase made from a mixture bound with a hydraulic binder. The aforementioned parameter can be improved by using a polymer modifier. The tests conducted at 0 °C demonstrated that the mixture with cement content of 2.0%, polymer modifier content of 2.0%, and bitumen emulsion content of 2.5% showed a higher fracture toughness K_IC_ than the subbase containing 2.0% of cement, 3.5% of polymer, and 5.0% of bitumen emulsion, i.e., 8.89 compared with 7.86. This also confirms the statement about replacing the bitumen emulsion with polymer. With a constant binder content equal to 2.0% of cement, the mixture containing 3.5% of polymer and 0.0% of bitumen emulsion demonstrates the same fracture toughness as the mixture with 0.5% of polymer and 5.0% of bitumen emulsion. The highest result was achieved by the mixture containing 3.5% of cement, 0.5% of polymer, and 2.5% of bitumen emulsion. The high cement content surely contributed to this result. Figure 8 below presents the convergence surfaces for the analysed parameter.

With no minimum polymer content in the mixture, the highest fracture toughness can be achieved for formulae containing more than 3.0% of cement and 2.5% of bitumen emulsion. An increase in the polymer content to 2.0% allows us to reduce the cement content in the mixture while maintaining a high fracture resistance. The addition of 3.5% of polymer enables a substantial reduction in the content of cement and bitumen emulsion contents, thereby allowing us to achieve a high fracture resistance of the recycled mixture even with 1.0% of emulsion and 2.0% of cement.

**Figure 9:**
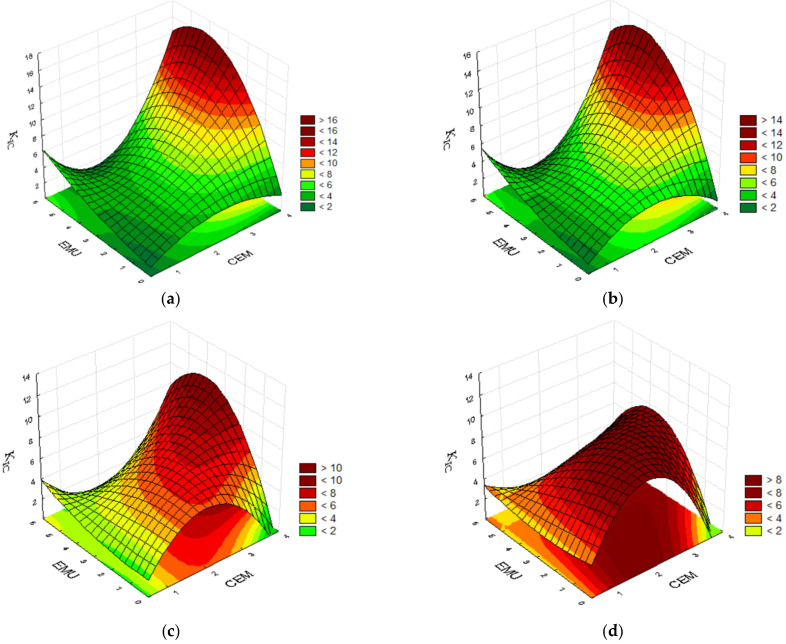
Plots for fracture toughness K_IC_ at the temp. of 20 °C, taking into consideration the cement and bitumen emulsion contents and the polymer emulsion content of (**a**) 0.0%; (**b**) 0.5%; (**c**) 2.0%; and (**d**) 3.5%.

When the test temperature is increased to 20 °C, it is possible to observe the significance of the mixture’s polymer content. The highest fracture toughness was achieved by the mixture containing 3.5% of cement, 0.5% of polymer, and 2.5% of bitumen emulsion. The subbase containing no bitumen emulsion, 2.0% of cement, and 3.5% of modifier achieved a very good result. Two mixtures achieved a similar fracture resistance of 7.4–7.7. The first mixture contains 3.5% of cement, 2.0% of polymer, and 5.0% of bitumen emulsion. The other mixture, which achieved a slightly better result, also contains 3.5% of cement, but its emulsion content was reduced to 2.5%, and its modifier content was increased to 3.5%. An optimisation of the recycled mixture’s composition is therefore very important. An adequate polymer modifier content allows the improvement of the fracture toughness and re-education in terms of the demand for bitumen emulsion in the mixture. Figure 9 below presents the convergence surfaces for the analysed parameter.

The mixture’s fracture toughness test conducted at 20 °C confirmed the high cement content’s contribution to obtaining the best results with no or minimum polymer content. The addition of 2.0% polymer modifier minimally lowers the obtained results of fracture toughness K_IC_, but makes it possible to reduce the cement and bitumen emulsion contents in the mixture. An increase in the polymer modifier content to 3.5% allows for an even greater reduction in the content of other binders in the mixture. Near maximum fracture toughness results are already obtained at 1.0% of cement content and 1.0% of bitumen emulsion content. It is worth noting that the maximum values of the aforementioned parameter are lower than for the subbase containing 0.5% of polymer, however they are more uniform for a broader range of component contents.

**Figure 10:**
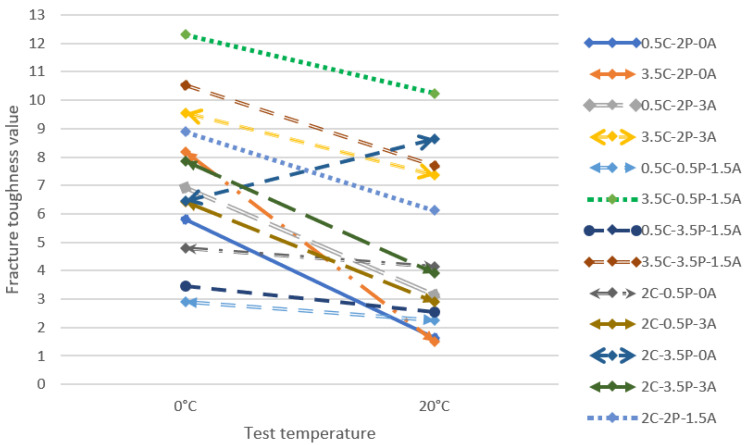
Plot of variation in fracture toughness K_IC_ depending on the test temperature.

Figure 10 below presents the plot for analysing the recycled mixtures’ fracture toughness K_IC_ and the variation in the parameter along with an increase in temperature.

There is a substantial impact of the designation temperature on the fracture toughness K_IC_. The mixtures with the highest results contained 3.5% of cement. However, it is worth noting that the 3.5C-2P-3A and 3.5C-3.5P-1.5A mixtures achieved very similar results. The analogous phenomenon occurs for the 3.5C-2P-3A and 2C-2P-1.5A mixtures. This means that adding polymer to the mixture reduces the risk of fracturing and enables a reduction in bitumen emulsion dosage without deteriorating the mixture’s properties. It must be stated that the 2C-3.5P-0A mixture achieved an increase in fracture toughness along with an increase in the test temperature.

## 5. Conclusions

The fracture toughness tests conducted in two temperatures provided more precise insight into the working nature of a recycled subbase containing a polymer modifier, and into the fracturing process. An analysis of the obtained results allows the formulation of the following conclusions:

1. The addition of a polymer modifier to a mineral–cement mixture containing bitumen emulsion achieves positive results for a broader range of cement and emulsion contents in the recycled mixture. It is not necessary to dose the maximum quantities of the aforementioned binders, i.e., above 4.0%, to obtain a high fracture resistance. With the 3.5% of modifier content, there is a real chance to reduce the cement and emulsion contents to nearly 1.0%.
2. The strain at maximum force ε_max_ parameter is twice as high when conducting tests at 20 °C, i.e., ε_max_ = 3.0, while for 0 °C, ε_max_ = 1.5. The addition of polymer to the mixture substantially improves the results while simultaneously reducing the bitumen emulsion content. This confirms the modifier’s contribution to ensuring the subbase’s higher strainability prior to fracturing.
3. The recycled subbase’s stress at break σ_max_ decreases along with an increase in its polymer content. It must be noted that an increase in the modifier content results in more constant stress, regardless of the emulsion and cement contents in the mixture. For a polymer content of 0.0%, σ_max_ = 0.1 ÷ 0.6, whereas for polymer content of 3.5%, the stress σ_max_ = 0.2 ÷ 0.3.
4. The recycled subbase’s stress at break σ_max_ is nearly twice as lower when comparing the results for the mixture with no polymer content to the mixture with a polymer content of 3.5%.
5. The recycled mixture’s polymer content of 2.0% already substantially reduces the occurring stress at break.
6. An increase in the recycled mixture’s polymer powder content contributes to more stable fracture toughness K_IC_ results. For polymer content of 0.0%, K_IC_ = 2.0 ÷ 14.0, whereas for polymer content of 2.0%, K_IC_ = 8.0 ÷ 14.0. The best results can be expected from mixtures with polymer contents of approx. 2.0%.

The conducted studies demonstrated that polymer modification reduces the fracturing in recycled subbases. This is extremely important from the point of view of pavement design engineers, investors, and users. It surely translates into higher pavement durability. Furthermore, adequate optimization of the composition of a recycled mixture containing polymer may contribute to the reduction in the demand for bitumen emulsion and cement. It can also contribute to financial savings on the aforementioned input materials. It therefore seems necessary to seek increasingly better solutions in the road industry. The modification of the road subbase composition made using the deep cold recycling technology with polymer analyzed in this paper can be a good example.

## Figures and Tables

**Table 1:** Properties of aggregate VA 0/31.5.

Property	Test	U.M.	Result	Category
Dimension d/D	EN 933-1 [29]	–	–	0/31
Particle size distribution	EN 933-1 [29]	–	–	GA_90_
Density	EN 1097-6 [30]	mg/m^3^	2.71	2.71
Shape index	EN 933-4 [31]	%	16.0	SI_25_
Flakiness index	EN 933-3 [32]	%	14.0	FI_25_
Percentage of crushed and broken surfaces	EN 933-5 [33]	%	98/2	C_90/3_
Frost resistance	EN 1367-1 [34]	%	3.4	F_1_
Resistance to fragmentation	EN 1097-2 [35]	%	23	LA_30_
Abrasion resistance	EN 1097-1 [36]	%	17.5	M_DE_25

**Table 2:** Properties of aggregate VA 0/4.

Property	Test	U.M.	Result	Category
Dimension d/D	EN 933-1 [29]	–	–	0/4
Particle size distribution	EN 933-1 [29]	–	–	G_F_85
Density	EN 1097-6 [30]	mg/m^3^	2.83	2.83

**Table 3:** Chemical composition of the EVA polymer [24].

Component	C	O	Mg	Si	Ca	Al
Share (%)	67.67	29.13	0.52	1.65	0.75	0.29

**Table 4:** Properties of Portland cement CEM I 42.5R.

Property	Test Method	Unit of Measure	Result
Initial setting time	EN 196-3 [38]	min	209
Compressive strength	EN 196-1 [39]	–	–
at 2 days		MPa	27.2
at 28 days		MPa	55.6
Soundness	EN 196-3 [38]	mm	0.8
Specific surface area	EN 196-6 [40]	cm^2^/g	3360

**Table 5:** Physical properties of the bitumen emulsion.

Property	Unit of Measure	C60B10ZM/R
Binder content	% (m/m)	60.0
Cement mixing stability	g	0.3
Sieve residue 0.5 mm	% (m/m)	0.06
Discharge time Ø 2 mm at 40°C	s	27
Adhesion to aggregate	%	75
Recycled bitumen penetration	0.1 mm	53
Recycled bitumen’s softening point	°C	55.2

**Table 6:** Test results.

Parameter	–	$\rho_{bssd}\;$	–	–	V_m_	–	Water Absorption (n_w_)			–	ITS_DRY_	–
Mixture	$\rho_{bssd}\;(\mathrm{mg}/m^{3})$	V (%)	σ	V_m_ (%)	V (%)	σ	n_w_ (%)	V (%)	σ	ITS_DRY_ (kPa)	V (%)	σ
0.5C-0.5P-1.5A	2.247	0.4	0.01	12.34	2.5	0.30	2.92	1.9	0.06	396.85	1.7	6.74
2.0C-0.5P-0.0A	2.293	0.3	0.01	12.58	2.1	0.26	3.98	3.6	0.14	643.94	2.3	14.72
2.0C-0.5P-3.0A	2.160	0.0	0.01	13.16	0.7	0.09	2.74	2.1	0.06	644.42	4.6	29.72
3.5C-0.5P-1.5A	2.263	0.7	0.02	10.45	2.8	0.29	1.62	6.9	0.11	1608.69	2.0	31.70
0.5C-2.0P-0.0A	2.239	0.4	0.01	9.78	4.1	0.40	3.43	3.2	0.11	363.95	2.6	9.60
0.5C-2.0P-3.0A	2.151	0.9	0.02	11.19	1.9	0.21	2.41	23.3	0.56	333.55	2.9	9.66
2.0C-2.0P-1.5A	2.200	0.8	0.02	12.45	5.5	0.70	2.81	12.9	0.36	714.88	3.2	22.86
3.5C-2.0P-0.0A	2.239	0.5	0.01	8.83	3.3	0.29	2.69	7.8	0.21	1370.25	3.6	48.87
3.5C-2.0P-3.0A	2.183	0.4	0.01	13.93	5.1	0.71	1.31	14.3	0.19	1163.31	1.2	13.66
0.5C-3.5P-1.5A	2.170	0.3	0.01	13.09	1.7	0.23	3.26	7.9	0.26	278.64	7.8	21.79
2.0C-3.5P-0.0A	2.203	0.5	0.01	13.55	3.0	0.41	3.48	2.2	0.08	591.55	2.3	13.77
2.0C-3.5P-3.0A	2.110	0.6	0.01	13.20	3.7	0.48	3.10	7.6	0.23	370.81	2.5	9.27
3.5C-3.5P-1.5A	2.180	0.8	0.01	12.15	5.8	0.71	2.02	6.4	0.13	1226.37	8.2	100.53

where: V (%)—coefficient of variation, and σ—standard deviations.

**Table 7:** Factor impact significance assessment.

Parameter	–	0 °C		20 °C	
–	Factor	Regressn Coeff.	p	Regressn Coeff.	p
	Mean/Interc.	1.14	0.0001	1.41	0.0001
	(1)CEM (L)	0.01	0.9814	0.25	0.1804
	CEM (Q)	−0.01	0.8427	0.02	0.5209
	(2)EMU (L)	−0.15	0.0199	−0.09	0.3154
ε_max_	EMU (Q)	0.02	0.0051	0.01	0.2629
	(3)RPP (L)	0.12	0.3515	−0.88	0.0001
	RPP (Q)	−0.02	0.4250	0.22	0.0001
	1L by 2L	0.01	0.3787	−0.02	0.2469
	1L by 3L	0.03	0.2307	−0.07	0.0421
	2L by 3L	−0.01	0.7424	0.07	0.0006
	Mean/Interc.	0.05	0.2193	−0.04	0.1594
	(1)CEM (L)	0.05	0.0563	0.12	0.0001
	CEM (Q)	0.01	0.2892	−0.01	0.0001
	(2)EMU (L)	0.02	0.2404	0.05	0.0001
σ_max_	EMU (Q)	−0.01	0.2635	−0.01	0.0001
	(3)RPP (L)	0.09	0.0033	0.03	0.1594
	RPP (Q)	−0.01	0.0146	0.01	0.0781
	1L by 2L	0.01	0.3868	0.01	0.0001
	1L by 3L	−0.01	0.1472	−0.01	0.0017
	2L by 3L	−0.01	0.8550	−0.01	0.0001
	Mean/Interc.	−0.64	0.4837	−0.96	0.2326
	(1)CEM (L)	2.59	0.0001	3.05	0.0001
	CEM (Q)	−0.08	0.4943	−0.42	0.0001
	(2)EMU (L)	1.14	0.0001	1.35	0.0001
K_IC_	EMU (Q)	−0.17	0.0001	−0.28	0.0001
	(3)RPP (L)	3.22	0.0001	0.55	0.2613
	RPP (Q)	−0.62	0.0001	0.23	0.0287
	1L by 2L	0.01	0.7948	0.29	0.0001
	1L by 3L	−0.26	0.0163	−0.31	0.0010
	2L by 3L	−0.01	0.8384	−0.23	0.0001

* Important experiment factors are underlined.

## Data Availability

The data presented in this study are available on request from the corresponding author.
